# Factors associated with enrollment, adherence, and retention in a remotely delivered tobacco cessation randomized controlled trial

**DOI:** 10.18332/tpc/216719

**Published:** 2026-06-30

**Authors:** Zhanette Coffee, Chiu-Hsieh Hsu, Christine E. Sheffer, Peter Giacobbi Jr., Kari M. Marano, Yessenya Barraza, Judith S. Gordon

**Affiliations:** 1 College of NursingUniversity of ArizonaTucsonUnited States; 2 Comprehensive Center for Pain and AddictionUniversity of ArizonaTucsonUnited States; 3 Mel and Enid Zuckerman College of Public HealthUniversity of ArizonaTucsonUSA; 4 Department of Health BehaviorRoswell Park Comprehensive Cancer CenterBuffaloUnited States; 5 College of Applied Human SciencesWest Virginia UniversityMorgantownUnited States; 6 School of Public HealthWest Virginia UniversityMorgantownUnited States

**Keywords:** adherence, tobacco, cessation, retention, enrollment

## Abstract

**Introduction:**

Remote tobacco cessation trials can expand access, yet enrollment, adherence, and retention remain challenging. This study aimed to identify individual- and neighborhood-level factors associated with enrollment, adherence, and retention in a two-arm, remotely delivered tobacco cessation randomized controlled trial (RCT). We conducted a secondary analysis of data from RCT NCT05277831.

**Methods:**

Adults who smoke were recruited between October 2022 and July 2024 through quitlines, recruiting firms, and community outreach conducted in Arizona, New York, and West Virginia. Participants were block-randomized to a behavioral cessation intervention or behavioral plus guided imagery intervention. Data were collected using online, self-reported questionnaires. Outcomes included enrollment (Session 1 completion), adherence (≥4/6 sessions), and 3 month retention, using multivariable logistic regression estimated adjusted odds ratios (AORs) for demographic, clinical, and neighborhood disadvantage covariates.

**Results:**

Of 1595 randomized participants, 1209 enrolled (75.8%), 914 (75.6%) were adherent, and 1046(86.5%) were retained at 3 months. The sample (mean age 50 years) was predominantly female (65%) and White (77%); about 35% had high school or lower education. Enrollment was lower among males (AOR=0.76; 95% CI: 0.60–0.97, p=0.03) those with lower education (AOR=0.74; 95% CI: 0.57–0.96, p=0.03), and those from more disadvantaged neighborhoods (AOR=0.99; 95% CI: 0.98–0.99, p=0.02). Adherence was higher with older age (AOR=1.02; 95% CI: 1.01–1.03, p=0.003), but lower with multiple household smokers (AOR=0.90; 95% CI: 0.82–1.00, p=0.05). Non-English primary language (AOR=0.36; 95% CI: 0.14–0.92, p=0.03), having more than one household smoker (AOR=0.88; 95% CI: 0.79–0.98, p=0.02), lack of a high school education (AOR=0.39; 95% CI: 0.23–0.65, p<0.001), and substance use (AOR=0.40; 95% CI: 0.2–0.81, p=0.01) were all associated with lower retention.

**Conclusions:**

Sociodemographic factors influenced enrollment, adherence, and retention, highlighting the importance of continued research to better understand and address engagement challenges in remotely delivered cessation studies.

## Introduction

Tobacco use continues to be the foremost cause of preventable illness, disability, and death in the United States (US), despite decades of public health efforts and widespread awareness of its harms^1^. An estimated 50.6 million, roughly one in five adults, currently use tobacco products^2^. In 2018, cigarette smoking cost the US more than $600 billion, including over $240 billion in direct healthcare spending and more than $372 billion in indirect economic losses, representing more than 15% of total US healthcare expenditures^3^. While quitting smoking offers profound and immediate health benefits, and several effective, evidence-based treatments exist, substantial obstacles persist^4^. These include limited access to effective care, inconsistent treatment outcomes, and low long-term cessation success. Only about 10% of people who attempt to quit smoking achieve sustained abstinence, even though two-thirds use evidence-based counseling and/or pharmacotherapy^5^. New therapeutic approaches are needed.

Randomized controlled trials (RCTs) are critical for testing new therapeutic approaches to improve tobacco cessation outcomes^6^. However, recruiting and retaining participants in cessation trials is becoming increasingly complex. A deeper understanding of the factors that influence enrollment, adherence, and retention is essential to improving engagement in cessation research^7^. Prior studies have identified several factors associated with enrollment and adherence in smoking cessation trials, including education level^6^, race and ethnicity^8^, type of intervention delivery^9^, and motivation to quit^10^. According to a literature review, adherence to smoking cessation medication regimens is also important^10^. In general, adherence to smoking cessation medication recommendations is linked to education level, race, co-occurring mental health conditions, side effects, and levels of nicotine dependence^10^. Furthermore, treatments that incorporate components of behavioral activation appear to increase protocol adherence^11^.

Remotely delivered trials can increase access to participation but face distinct barriers and challenges in maintaining participant engagement^12,13^, including limited digital literacy and inconsistent technology access. A lack of in-person contact can also serve as a barrier to communication, trust, accountability, and treatment outcomes in remotely delivered studies^13^.

Emerging research suggests that neighborhood-level socioeconomic factors may also influence study enrollment, adherence, and retention. For instance, the Area Deprivation Index (ADI), a validated measure of neighborhood-level socioeconomic disadvantage^14^, has been associated with higher tobacco use prevalence rates^15^ and disparities in access to cessation resources^16^. Many people with social disadvantages face additional barriers to study participation, including limited health literacy, reduced technology access, and competing socioeconomic demands^17,18^.

The *Be Smoke Free* study (R01AT011500; PI: J. Gordon) is a large, remotely conducted pragmatic RCT that compared the effectiveness of an integrative guided imagery (GI) intervention combined with behavioral counseling, in comparison to a standard behavioral treatment alone for smoking cessation^4^. The *Be Smoke Free* Study was conducted entirely remotely (all contact was done via phone or online), including recruitment, consent, baseline, data collection, intervention delivery, and follow-up data collection. Outcomes from any RTC should be viewed in the context of study enrollment, adherence, and retention. There are complexities in conducting an entirely remotely delivered pragmatic RCT that affect enrollment, adherence, and retention. Therefore, the study reported herein was focused on *Be Smoke Free* study factors associated with study enrollment, adherence, and retention. The goal of this study was to inform future remote tobacco cessation trial designs and methods to maximize their rigor and reproducibility.

## Methods

### Design

This study is a secondary analysis of data collected from a two-arm pragmatic RCT that evaluated the efficacy of a GI intervention compared to an evidence-based behavioral treatment.

### Data source and permissions

Data for this analysis were drawn from the *Be Smoke Free* randomized controlled trial conducted by Gordon et al.^4^ between October 2022 and July 2024 (ClinicalTrials.gov Identifier: NCT05277831; International Registered Report Identifier [IRRID]: PRR1-10.2196/48898). Funded by the National Center for Complementary and Integrative Health (NCCIH) in September 2021 (R01AT011500; PI: J. Gordon), the study was originally approved on April 9, 2021 and the most recent approval was on December 3, 2025 by the University of Arizona Institutional Review Board (#2103633455). All participants provided informed consent.

Participants were recruited from three states (Arizona, New York, and West Virginia) using two approaches: 1) referrals from collaborating state quitlines; and 2) population-based recruitment methods such as social media posts, local networks, and a professional recruitment firm^4^. Eligible participants were adults who currently smoked and resided in one of the three target states. Participants were stratified by state and recruitment method, then randomized using Research Electronic Data Capture (REDCap) via a block randomization table generated by the study biostatistician. Participants were assigned to either a behavioral intervention (B) or a guided imagery (GI) intervention. Participants in the B intervention received a previously validated cognitive/behavioral intervention that included six sessions (e.g. reasons for quitting, triggers for smoking, dealing with cravings, preventing relapse, etc) focused on identifying and changing smoking-related cognitions and behaviors^4^. Participants in the GI intervention received the same components as those in the B intervention, plus they created a guided imagery script in each session, with assistance from their study coach, which was recorded as an audio file and sent to the participant to listen to each day between sessions. The purpose of the GI audio file was to reinforce motivation and changes to smoking-related cognitions and behaviors^4^. Participants in both conditions received up to 4 weeks of nicotine patches and/or lozenges^4^. Validated questionnaires from prior research^5,19^ were used to collect study measures. Participants also received a $15 gift card for completing the survey at 3 months.

### Inclusion and exclusion criteria

Inclusion criteria for the *Be Smoke Free* study were as follows: participants had to report cigarette smoking as their primary form of tobacco use; smoke daily for the past 30 days; be at least 18 years old; speak English; reside in Arizona, New York State, or West Virginia; own a smartphone with SMS text messaging capability; agree to receive telephone counseling and SMS reminders; and be able to download and access MP3 or MP4 audio files.

Exclusion criteria were: lack of phone or text messaging capability; non-English speakers; more than one participant per household; diagnosis of psychosis; current participation in any tobacco cessation program or use of cessation medications; prior use of quitline services within the past 12 months; or significant cognitive impairment due to dementia, traumatic brain injury, or other causes affecting memory and comprehension.

### Variables of interest

#### Enrollment

In this study, interested individuals were screened for eligibility by trained study staff via telephone or through REDCap. Eligible participants completed informed consent and a baseline survey either by phone or electronically through REDCap before being randomized into the study. Written informed consent was obtained electronically and securely stored in REDCap. After randomization, participants received program materials specific to their assigned treatment condition. Individuals found to be ineligible were referred back to their state’s quitline through a warm handoff process, where study staff contacted quitline enrollment staff and directly transferred the caller, or through a HIPAA-compliant method, such as encrypted email. Once randomized, we attempted to schedule participants for an initial treatment visit (Session 1). Participants who were scheduled and completed Session 1 were considered ‘enrolled’ and received follow-up surveys. We experienced a challenge between randomization and initiating the first treatment session, potentially due to the remote nature of the trial. We experienced 24.2% attrition between randomization and Session 1. The main reason (57%) was being unable to contact participants to schedule a session. It was likely that many of these randomized participants completed the baseline assessment solely to receive the $10 gift card. Due to the high attrition, the study team determined that there was not sufficient power to test the study’s hypotheses that the addition of guided imagery to behavioral treatment would be more effective than behavioral treatment alone, because almost a quarter of randomized participants received no treatment at all. Therefore, with permission from NCCIH, we continued recruitment until we enrolled the originally proposed sample size of 1200. Thus, ensuring that we would have sufficient statistical power to test the effectiveness of guided imagery plus behavioral treatment versus behavioral treatment alone.

#### Adherence

Adherence was defined as attendance in at least four of six scheduled treatment sessions.

#### Retention

Retention was defined as completion of the assessment at 3 months.

#### Participant demographic information

Quantitative, self-reported, sociodemographic information was collected via an online baseline questionnaire from participants. Variables included: age (18–99 years), gender (male, female, other), education level (<high school, high school degree or GED, some college, college degree, advanced degree), primary language (English, Spanish, English and Spanish, English and Other, Other), number of household smokers (1–10), marital status (single/never married, married, married but separated, divorced, widowed), insurance (Medicare, Medicaid, state insurance marketplace, employer, other, none), mood disorder (yes, no, don’t know), anxiety (yes, no, don’t know), substance use (yes, no, don’t know), cannabis use (smoke cannabis or marijuana or CBD that contains THC, vape cannabis or marijuana or CBD that contains THC, edible cannabis or marijuana or CBD that contains THC, other cannabis product, I do not use any cannabis products), and state (AZ, NY, WV). The focus of this work was to identify the variables associated with enrollment, adherence, and retention, not on evaluating the association between a variable of interest and enrollment, adherence, and retention. Therefore, all variables were examined, and no specific variables were considered as confounders.

#### Area Deprivation Index (ADI)

The Neighborhood Atlas uses the Area Deprivation Index (ADI) to measure neighborhood disadvantage^14^. For our sample, an R function, *calculate_adi*, in the library of *sociome* was used to calculate raw ADI scores by performing factor analysis using 2023 American Community Survey (ACS) data. The raw ADI scores were then standardized to have a mean of 100 and a standard deviation of 20. Using the entire 2023 ACS data, the standardized ADI scores ranged from 16.76 to 230.98. Based on previous research, we dichotomized the standard ADI scores into ≥ or <75th percentile (111.25)^20-22^.

### Data analysis

Participant characteristics were summarized using mean±standard deviation and median with interquartile range (IQR) for continuous variables, and frequency and percentage for categorical variables by enrollment/adherence status (defined as completing at least four sessions). Language was dichotomized into English versus all other languages, ensuring that all subsequent comparisons reflected differences between English speakers and non-English participants. Wilcoxon rank-sum tests were performed to compare continuous variables between groups. Fisher’s exact tests were performed to compare categorical variables between groups. Logistic regression was performed to identify the variables associated with enrollment, adherence, and 3 month retention, respectively, where the variables with a univariate analysis p<0.05 were included in the adjusted (multivariable) analysis. Multicollinearity was assessed by the variance inflation factor, and the Hosmer-Lemeshow test was conducted to assess the predicted probabilities. Multiple Imputation by Chained Equations (MICE) was performed to handle missing data. MICE was based on 15 imputations. The analysis based on the imputed datasets was considered a sensitivity analysis. Statistical analysis was performed using R 4.4.0.

## Results

### Participant characteristics

Among the n=1595 randomized participants, n=1209 (75.8%) were enrolled, n=914 (75.6%) were adherent (completed ≥ 4 sessions), and n=1046 (86.5%) were retained at 3 months (Table 1).

**Table 1:** Participant characteristics of adults in a remotely delivered randomized controlled trial, Arizona, New York, and West Virginia, 2022–2024 (N=1595)

Variables	Randomized (N=1595) n (%)	Enrolled (N=1209) n (%)	Adherent (N=914) n (%)	3 month retention (N=1046) n (%)
**Age** (years), mean±SD, median (IQR)	50.43±12.29 51.00 (41.00–60.00)	50.82±12.18 52.00 (41.00–60.00)	51.77±12.40^b^ 53.00 (42.00–61.00)	50.96±12.42 52.00 (41.00–60.00)
**Age>50 years**	812 (50.91)	635 (52.52)^a^	517 (56.56)	554 (52.96)
**Gender**				
Male	563 (35.30)	409 (33.83)	313 (34.25)	354 (33.84)
Female	1018 (63.82)	789 (65.26)	592 (64.77)	681 (65.11)
Trans/nonbinary/nonconforming	10 (0.63)	9 (0.74)	7 (0.77)	9 (0.86)
Prefer not to answer	4 (0.25)	2 (0.17)	2 (0.22)	2 (0.19)
**Race**				
White	1224 (76.74)	934 (77.25)	700 (76.59)	802 (76.67)
Black/African American	192 (12.04)	140 (11.58)	113 (12.36)	128 (12.24)
Asian	20 (1.25)	16 (1.32)	14 (1.53)	15 (1.43)
Hawaiian/Pacific Islander	4 (0.25)	1 (0.08)	1 (0.11)	1 (0.10)
American Indian/Alaska Native	36 (2.26)	29 (2.40)	21 (2.30)	26 (2.49)
Other	64 (4.01)	46 (3.80)	39 (4.27)	39 (3.73)
Multiracial	76 (4.76)	58 (4.80)	43 (4.70)	53 (5.07)
Don't know	5 (0.31)	4 (0.33)	4 (0.44)	4 (0.38)
Prefer not to answer	37 (2.32)	30 (2.48)	20 (2.19)	24 (2.29)
**Hispanic**				
No	1396(87.52)	1061(87.76)	804 (87.96)	917 (87.67)
Yes	169 (10.60)	124 (10.26)	93 (10.18)	107 (10.23)
Prefer not to answer	30 (1.88)	24 (1.99)	17 (1.86)	22 (2.10)
**Education level**				
Lower than high school	105 (6.58)	74 (6.12)	53 (5.80)	62 (5.93)
High school/general educational development	448 (28.09)	317 (26.22)	221 (24.18)	267 (25.53)
Some college/university	604 (37.87)	462 (38.21)	347 (37.96)	398 (38.05)
College/university	333 (20.88)	267 (22.08)	219 (23.96)	241 (23.04)
Advanced	87 (5.45)	76 (6.29)	62 (6.78)	67 (6.41)
Prefer not to answer	18 (1.13)	13 (1.08)	12 (1.31)	11 (1.05)
**Primary language**				
English	1543 (96.74)	1172(96.94)	882 (96.50)	1017(97.23)^a^
Spanish	3 (0.19)	2 (0.17)	2 (0.22)	2 (0.19)
English and Spanish	26 (1.63)	18 (1.49)	14 (1.53)	14 (1.34)
English and Other	14 (0.88)	10 (0.83)	9 (0.98)	6 (0.57)
Other	4 (0.25)	3 (0.25)	3 (0.33)	3 (0.29)
Prefer not to answer	5 (0.31)	4 (0.33)	4 (0.44)	4 (0.38)
**Number of people who smoke in household**, mean±SD, median (IQR)	1.42±1.56 1.00 (1.00–2.00)	1.43±1.68 1.00 (1.00–2.00)	1.34±0.77 1.00 (1.00–2.00)	1.36±0.75 1.00 (1.00–2.00)
Missing	235	172	128	144
>1	428 (31.47)	329 (31.73)	244 (31.04)^a^	287 (31.82)^a^
**Marital status**				
Single/never married	546 (34.23)	401 (33.17)	293 (32.06)	339 (32.41)
Married	384 (24.08)	314 (25.97)	245 (26.81)	276 (26.39)
Married but separated	104 (6.52)	71 (5.87)	52 (5.69)	57 (5.45)
Divorced	398 (24.95)	300 (24.81)	222 (24.29)	258 (24.67)
Widowed	130 (8.15)	96 (7.94)	79 (8.64)	93 (8.89)
Prefer not to answer	33 (2.07)	27 (2.23)	23 (2.52)	23 (2.20)
**Health insurance**				
Medicare	995 (62.38)	761 (62.94)	562 (61.49)	654 (62.52)
Medicaid	648 (40.63)	472 (39.04)	343 (37.53)	408 (39.01)
Through state health	130 (8.15)	100 (8.27)	75 (8.21)	84 (8.03)
Through employer	313 (19.62)	261 (21.59)	209 (22.87)	226 (21.61)
Other insurance	147 (9.22)	117 (9.68)	86 (9.41)	99 (9.46)
No insurance	160 (10.03)	126 (10.42)	90 (9.85)	110 (10.52)
**Area Deprivation Index (ADI)**, mean±SD, median (IQR)	103.83±17.60 105.39 (94.13–114.35)	103.02±17.87^a^ 104.84 (93.11–113.85)	102.16±18.41 103.93 (91.63–113.14)	102.88±18.10 104.87 (92.38–113.85)
Missing	19	12	7	10
≥ 75th percentile	532 (33.76)	378 (31.58)	267 (29.44)	327 (31.56)
**Education≤High school**	553 (34.67)	391 (32.34)^a^	274 (29.98)	329 (31.45)^b^
**Health status**				
Asthma	284 (17.81)	216 (17.87)	155 (16.96)	187 (17.88)
Chronic obstructive pulmonary disease	282 (17.68)	203 (16.79)	159 (17.40)	175 (16.73)
Diabetes mellitus	194 (12.16)	145 (11.99)	115 (12.58)	133 (12.72)^a^
Heart disease	130 (8.15)	96 (7.94)	80 (8.75)	82 (7.84)
Hypertension	436 (27.34)	331 (27.38)	263 (28.77)	295 (28.20)
Cancer	51 (3.20)	38 (3.14)	27 (2.95)	30 (2.87)
Mood	412 (25.83)	309 (25.56)	231 (25.27)	267 (25.53)
Anxiety	606 (37.99)	460 (38.05)	346 (37.86)	404 (38.62)
**Substance use**	135 (8.46)	102 (8.44)	67 (7.33)	79 (7.55)^a^
**Regularly use cannabis**	465 (29.15)	351 (29.03)	264 (28.88)	301 (28.78)
**State**				
New York (NY)	661 (41.44)	512 (42.35)	419 (45.84)	438 (41.87)
Arizona (AZ)	572 (35.86)	441 (36.48)	321 (35.12)	384 (36.71)
West Virginia (WV)	362 (22.70)	256 (21.17)	174 (19.04)	224 (21.41)
**Group**				
Behavioral	798 (50.03)	633 (52.36)	472 (51.64)	542 (51.82)
Guided Imagery (GI)	797 (49.97)	576 (47.64)	442 (48.36)	504 (48.18)

IQR: interquartile range.

a p<0.05

b p<0.01.

Of those participants randomized, the mean age was 50 years (SD=12.29). About two-thirds were female (64%) and more than three-quarters were White (77%). About one-third (35%) had a high school education or lower, and over one-third (38%) had some college. English was the primary language for greater than 97% of participants. About 32% reported living with more than one person who smoked in the household, and about one-third were single/never married. The most common health insurances were Medicare (62%) and Medicaid (40%); 10% reported no insurance. About one-third (34%) lived in neighborhoods at or above the 75th percentile of the ADI. Overall, participants lived in neighborhoods reflecting moderate socioeconomic disadvantage (median ADI=105.39, IQR: 94.13–114.35).

Common comorbidities included anxiety (38%), mood disorders (25%), hypertension (27%), COPD (17%), asthma (18%), and diabetes (12%). Substance use disorder was reported by 8%, and 29% reported regular cannabis use. Geographically, participants were drawn from New York (42%), Arizona (36%), and West Virginia (21%).

### Variables associated with enrollment

Several factors were associated with enrollment. As shown in (Table 2), gender was a consistent predictor: males had significantly lower odds of enrollment overall (AOR=0.76; 95% CI: 0.60–0.97, p=0.03). ADI was inversely associated with enrollment (AOR=0.99; 95% CI: 0.98–1.00, p=0.02). In addition, participants with lower than a high school education had reduced odds of enrollment (AOR=0.74; 95% CI: 0.57–0.96, p=0.03). Other demographic variables (race/ethnicity, marital status, household smoking, chronic conditions, insurance, and cannabis use) were not significantly associated with enrollment. For additional enrollment details, please refer to the Supplementary file Figure 1 (CONSORT diagram).

**Table 2:** Multivariable analysis of enrollment (N=1209), adherence (N=914), and 3-month retention (N=1046) in a remotely delivered randomized controlled trial, Arizona, New York, and West Virginia, 2022–2024

Variables	Enrollment		Adherence		3 month retention	
	AOR (95% CI)	p	AOR (95% CI)	p	AOR (95% CI)	p
Age (years)	1.01 (1.00–1.02)	0.18	1.02 (1.01–1.03)	0.003		
Male	0.76 (0.60–0.97)	0.03				
Primary language: Other vs English only					0.36 (0.14–0.92)	0.03
Number of household smokers: >1 vs 1			0.9 (0.816–1.00)	0.05	0.88 (0.79–0.98)	0.02
Area Deprivation Index (ADI)	0.99 (0.98–1.00)	0.02	0.99 (0.98–1)	0.06		
No High school	0.74 (0.57–0.96)	0.03	0.75 (0.55–1.03)	0.08	0.39 (0.23–0.65)	<0.001
Substance use			0.63 (0.39–1.02)	0.06	0.40 (0.2–0.81)	0.01

AOR: adjusted odds ratio.

To assess potential multicollinearity in the multivariable models, variance inflation factors (VIFs) were examined for all covariates. Across the enrollment, adherence, and retention models, VIF values were uniformly low, generally ranging from approximately 1.0 to 1.3, indicating no evidence of problematic multicollinearity among the variables included in the adjusted analyses.

### Adherence by completed session

**Figure 1:**
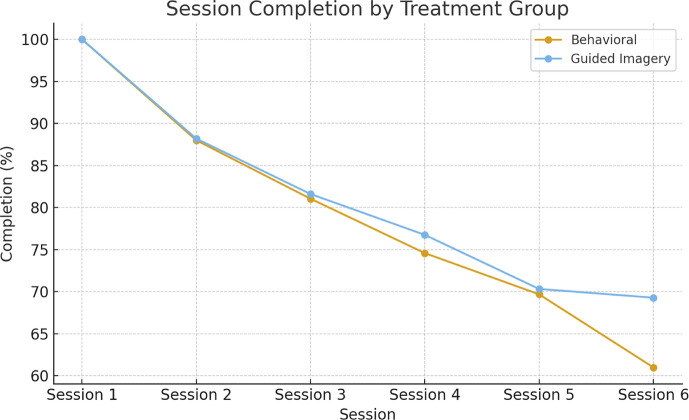
Session-specific completion rates by treatment group, remotely delivered randomized controlled trial, Arizona, New York, and West Virginia, 2022–2024 (N=914)

Figure 1 shows the adherence percentage of completion rates by group. Adherence, defined as completion of at least four sessions, was achieved by 75% of participants in the Behavioral arm (n=472) and 77% in the GI arm (n=442). No significant differences were found between groups. Session-specific completion declined steadily over time in both groups, and attrition patterns were similar.

### Variables associated with adherence

As displayed in Table 2, older age was positively associated with adherence (AOR=1.02; 95% CI: 1.01–1.03, p=0.003). Household smoking was inversely associated with adherence, with participants from households with more than one smoker showing reduced odds of adherence (AOR=0.90; 95% CI: 0.82–1.00, p=0.05). No other variables were significantly associated with adherence.

### Variables associated with retention

As shown in Table 2, predictors of significantly lower 3 month retention included: primary language (i.e. those primarily speaking a non-English language) (AOR=0.36; 95% CI: 0.14–0.92, p=0.03); having more than one household smoker (AOR=0.88; 95% CI: 0.79–0.98, p=0.02); having lower than a high school education (AOR=0.39; 95% CI: 0.23–0.65, p<0.001); and reporting other substance use (AOR=0.40; 95% CI: 0.2–0.81, p=0.01). No other variables were significantly associated with retention.

## Discussion

This study identified factors associated with enrollment, adherence, and 3 month retention in a remotely delivered smoking cessation treatment trial. Overall, strong enrollment, adherence, and retention rates suggest high participant engagement with treatment and study procedures, as well as robust data quality and completeness, especially for a remotely delivered study. Enrollment, adherence, and retention rates can impart important information about the study methodology, execution, and ultimately the validity and generalizability of study findings. Several factors emerged as consistently associated with these important aspects of this study.

Women were more likely to enroll after randomization, as were those with higher socioeconomic status, including higher education. Although the proportionate differences in enrollment were relatively small in this study, the findings are consistent with other studies^10,23^ highlighting persistent challenges in engaging men, people of lower socioeconomic status, and people with lower level of education in smoking cessation treatment trials^24^. Similar findings from existing literature suggest that neighborhood disadvantage may create structural barriers to participation, even in remotely delivered interventions^25^. This is especially important because people of lower socioeconomic status are more likely to smoke cigarettes^26^.

Area Deprivation Index (ADI) or socioeconomically disadvantaged neighborhood was statistically associated with enrollment. However, the per-unit odds ratio was small, suggesting a modest association at the individual level, consistent with prior work examining socioeconomic status in research participation^24^. Neighborhood disadvantage may limit access, readiness, or sustained participation, even in virtual formats designed to reduce geographical barriers^16^. Even modest socioeconomic disadvantage may meaningfully shape enrollment patterns at the population level, underscoring the importance of incorporating targeted outreach and structural supports into the design of future remotely delivered trials to promote equitable participation.

Adherence was statistically associated with older age. However, the magnitude of this effect was small and may have limited practical significance. This finding is consistent with prior literature suggesting that older age plays a modest role in sustaining engagement in smoking cessation treatments^10,27^. The findings from this remotely delivered study suggest that the older people who participated in this study were able to effectively engage with and navigate the use of smartphones and the Internet required by the study. Perhaps the use of a telephone-delivered treatment and study protocols and the limited use of the Internet enabled older participants to comfortably engage in the study. Moreover, adherence rates in our study compare favorably with those reported in other remotely delivered cessation trials^27^, reinforcing the feasibility of technology-based approaches for promoting sustained engagement among older populations.

In contrast, multiple household smokers were associated with lower adherence, suggesting that both environmental and health-related factors can undermine sustained participation. Because this study was delivered remotely, participation required the use of a mobile phone for counseling sessions, text reminders, and completing surveys, which may have presented additional challenges for individuals with limited phone plans or jobs that did not allow for regular participation during study hours.

Lower rates of adherence were found among participants who lived with other people who smoke, aligning with previous research demonstrating that exposure to household smoking can negatively influence engagement and tobacco cessation program adherence^28^. These results reinforce the need for smoking cessation treatment studies to incorporate strategies that address household smoking environments.

Three-month retention was shaped by several sociodemographic factors. Substance use was associated with lower retention, which is consistent with previous studies demonstrating that concurrent or past alcohol or drug use can complicate engagement in cessation programs and reduce treatment effectiveness^29,30^. However, evidence shows that quitting smoking increases the odds of substance use disorder recovery^31^, suggesting that smoking cessation itself may serve as a valuable tool for supporting broader recovery goals among the millions of US adults living with a current substance use disorder.

Participants whose primary language was other than English had lower retention, suggesting potential gaps in linguistic or cultural accessibility that may hinder sustained engagement. This aligns with prior research showing that people who smoke whose primary language is not English often require linguistically tailored or mobile cessation supports to maintain engagement, and that culturally mismatched interventions can undermine retention^32^.

Household environment also played a meaningful role in retention, as having more than one household smoker was associated with decreased retention. This finding aligns with prior research indicating that individuals who were sometimes around other smokers were nearly twice as likely to opt out of a cessation study within the first 3 days of a quit attempt^33^, suggesting that smoking in one’s immediate environment may negatively influence early engagement.

Education similarly emerged as a predictor of retention, with individuals lacking a high school education showing lower odds of remaining engaged in the study. This pattern is consistent with studies indicating that a lower level of education is associated with poor cessation outcomes and lower engagement with both behavioral and pharmacologic interventions^34^.

Furthermore, both the GI and the standard treatments incorporated self-directed ‘homework’ components (e.g. reading a guidebook in the standard group and practicing guided imagery exercises in the GI group), which may have been more challenging for participants with less experience with structured independent learning. Our findings align with prior research indicating that people with higher education are more likely to engage in treatment^35^, and higher education is also linked to greater harm perception of smoking compared to lower level of education^29^. This underscores the importance of applying a social determinants of health framework in tobacco cessation to address education-related disparities.

Furthermore, other substance use predicted lower retention. Tobacco use commonly overlaps with other substance use and mental health conditions^36^, and lower retention among participants reporting substance use in our study is consistent with prior evidence showing that individuals with co-occurring conditions have poorer retention in smoking cessation programs^30^.

The findings from this study highlight the importance of considering both individual- and context-level factors in designing and conducting remote tobacco cessation trials. Socioeconomic disadvantage, education level, household smoking environments, and substance use may represent factors that influence participation and warrant further investigation. Smoking cessation treatment studies programs should prioritize targeted outreach to disadvantaged and underrepresented groups, adapt intervention content for participants with lower level of education, encourage household-level support for behavior change, design interventions for social groups of people who smoke (e.g. couples, peer groups, etc), and address issues related to other substance use and recovery to optimize treatment engagement. Developing support strategies addressing digital access and competing socioeconomic demands may be necessary to enhance engagement among individuals from highly disadvantaged communities. Even in virtual formats, disparities in digital literacy, limited internet access, and competing life demands can hinder participation.

### Limitations

This study utilized data from a pragmatic RCT with defined inclusion and exclusion criteria, which may limit the generalizability of findings to broader populations. We acknowledge that the sample was predominantly female and White, and participation required smartphone ownership, which may also limit generalizability. However, over a third of the participants lived in a socioeconomically disadvantaged neighborhood, and more than a third had a high school or lower education. In addition, participants received a $15 gift card for completing the follow-up survey at 3 months, which may have contributed to the high retention observed in this study. However, the modest value of the incentive for a 15–20 min survey suggests it likely had only a modest effect on retention. Nevertheless, the use of incentives should be considered when interpreting retention outcomes, which may limit generalizability to research where such incentives are not available. As the analyses were exploratory and not designed to test a specific a priori hypothesis, a power analysis was not conducted. In addition, we were unable to track individuals who were randomized but ultimately did not enroll in the study, preventing us from confirming their reasons for non-participation. As a result, residual confounding variables may have influenced enrollment, adherence, and retention outcomes. As a secondary analysis focused on identifying associations rather than testing causal relationships, we cannot draw definitive conclusions about the underlying causes of non-enrollment or non-adherence. While ADI captured neighborhood disadvantage, we lacked individual-level socioeconomic measures (income, employment, housing instability) that could refine mechanistic understanding. Finally, the reliance on self-reported questionnaire data may introduce information bias and potential misclassification, which should be considered when interpreting the findings.

### Implications for practice and trial design

Findings from this study suggest that sociodemographic and contextual variables may be important considerations for understanding engagement in clinical trials. Future research may explore whether targeted outreach approaches for men, individuals with lower level of education, and those living in socioeconomically disadvantaged neighborhoods could improve enrollment, adherence, and retention. Enhancing adherence may require additional support for participants with multiple household smokers, while targeted retention strategies could benefit populations at higher risk of disengagement, such as those with lower level of education, multiple household smokers, or substance use disorder. Integrating new approaches into remotely delivered cessation programs has the potential to improve generalizability and improve long-term outcomes.

## Conclusions

This study highlights key sociodemographic and contextual variables associated with engagement in a remotely delivered tobacco cessation program. These findings suggest that future research should further examine whether approaches such as targeted outreach or additional supports for groups at higher risk of disengagement may influence enrollment, adherence, and retention. Continued investigation of these variables and environmental factors is important for informing the design and methods of remotely delivered tobacco cessation trials, and to better understand how social determinants may shape participation in remotely delivered tobacco cessation interventions.
